# The role of illness perception in the physical activity domain of health-promoting lifestyle among patients with non-communicable diseases: A systematic review

**DOI:** 10.1371/journal.pone.0311427

**Published:** 2024-11-08

**Authors:** Sharifah Maziah Syed Shamsuddin, Norfazilah Ahmad, Mohd Firdaus Mohd Radi, Roszita Ibrahim

**Affiliations:** 1 Department of Public Health Medicine, Faculty of Medicine, Universiti Kebangsaan Malaysia, Cheras, Kuala Lumpur, Malaysia; 2 Medical Development Division, Ministry of Health, Putrajaya, Malaysia; Taipei Medical University, TAIWAN

## Abstract

**Background:**

The global mortality caused by non-communicable diseases is on the rise. Health-promoting lifestyles are among the most effective approaches, yet the physical activity domain consistently scores the lowest. Physical activity is linked to individual behaviour and influenced by numerous factors. Illness has been identified as a key factor in behavioural change. Therefore, the primary purpose of this review was to investigate the role of illness perception in the physical activity domain among patients with non-communicable disease.

**Method:**

This systematic review follows the Preferred Reporting Items for Systematic Reviews and Meta-Analyses 2020 guidelines. A literature search was conducted across three scientific databases (Scopus, PubMed, and Web of Science), targeting original articles published in English between 2014 and 2024. The quality of the eligible articles was assessed using the Joanna Briggs Institute Critical Appraisal tools. The findings were synthesised through content analysis.

**Results:**

A total of 17 studies were included, identifying both the direct and indirect effects of illness perception variables as a whole or in their respective dimensions. The illness perception variable has demonstrated a significant positive and negative relationships with the physical activity domain.

**Limitation:**

The majority of the included studies had a cross-sectional design. Therefore, the evidence quality was relatively low and exhibited a high risk of bias. Furthermore, there was language bias as only English-language publications were selected.

**Conclusion:**

The findings of this review will serve as a guide for healthcare providers in enhancing physical activity adherence among patients with non-communicable diseases through an illness perception approach. This approach can be integrated into clinic consultations and intervention programmes. Future studies are warranted to evaluate the effectiveness of the illness perception approach in promoting physical activity adherence.

## Introduction

The prevalence of non-communicable diseases (NCDs) varies across different populations and regions. Globally, in 2016, NCDs accounted for 71% (40.5 million) of deaths [1]. Over recent decades, this prevalence has risen to 74% (41 million) [2]. The main types of NCDs include cardiovascular disease, cancer, diabetes, and chronic respiratory disease. Among these, cardiovascular disease contributed to the highest number of NCD deaths annually (17.9 million people annually), followed by cancer (9.3 million) and diabetes (2.0 million) [2].

The risk factors for NCD can be divided into non-modifiable and modifiable factors. Non-modifiable factors include age, gender, and family history. Modifiable factors are associated with unhealthy lifestyles, such as physical inactivity, unhealthy diet, smoking, and excessive alcohol consumption [3]. Lifestyle encompasses an individual’s or group’s chosen way of living style, including physical activities, leisure time, sleep patterns, interpersonal relationships, spirituality, safety practices, and nutrition habits [4]. One of the effective strategies in NCD prevention and management is promoting a healthy lifestyle intervention programme [5]. This can be achieved through health-promoting lifestyle approach.

Health-promoting lifestyles have demonstrated positive impacts on health outcomes [6], NCD prevention [7–9], and the reduction of healthcare costs [10, 11]. A health-promoting lifestyle is defined as a multidimensional concept that is contributed by self-initiated action, various levels of behaviour, and self-perception by individuals to maintain or improve their wellness level, self-actualisation, and individual fulfilment [12]. It encompasses six domains: i) spiritual growth, ii) stress management, iii) nutrition, iv) interpersonal relationship, v) health responsibility, and vi) physical activity [13].

Physical activity covers all forms of body movement, such as walking, cycling, wheeling, sports, active recreation, and play [14]. Despite its known benefits in reducing the risk of NCDs, a previous study found that a significant portion of the global population (31.1%) remains physically inactive [15]. The majority of health-promoting lifestyle studies reported that the physical activity domain demonstrated the lowest mean domain score among various study populations of the general community [13, 16], medical undergraduates [17, 18], nurses [19], and older persons with NCDs [20]. Several factors influence physical activity adherence among NCD patients, including age, gender, socioeconomic status, education level, and illness perception [21].

Illness perception significantly influences physical activity participation, capacity, and adherence among individuals with various types of NCDs. It is defined as “an attempt by patients to give meaning and to understand their illness at a cognitive as well as emotional level” [22]. Illness perception comprises individuals’ representations of their illness, including beliefs about its causes, potential effects, and manageability [23]. Two widely used quantitative measures for assessing illness perception are the Brief Illness Perception Questionnaire (BIPQ) and the Revised Illness Perception Questionnaire (IPQ-R), both of which have been validated in multiple languages for various study populations. There are nine dimensions measured through BIPQ including; (i) Consequences, (ii) Timeline, (iii) Personal control, (iv) Treatment control, (v) Identity, (vi) Concern, (vii) Emotional representations, (viii) Illness coherence and (ix) causal attribution of the illness or disease [24]. Notably, item nine prompts participants to list the three most likely causes of their disease. On the other hand, the IPQ-R measures 12 dimensions which are; (i) Timeline acute/chronic, (ii) Timeline cyclical, (iii) Consequences, (iv) Personal control, (v) Treatment control, (vi) Emotional representations, and (vii) Illness coherence, (viii) Identity, (ix) Psychological attributions, (x) Risk factor attributions, (xi) Immune attributions, and (xii) Chance attributions [25].

Illness perception significantly influences physical activity through both direct and indirect effects [26, 27]. This finding has been demonstrated in previous studies conducted among different population backgrounds, types, and nature of the NCD [26, 28, 29]. Therefore, the illness perception factor based on the Leventhal self-regulation model is a potential approach to be integrated into the NCD intervention program through education and counselling sessions during clinic visits [30]. The intervention programme may focus on five dimensions identity, cause, timeline, consequences, and controllability. Therefore, it is warranted to explore the role of illness perception towards physical activity participation and adherence among NCD patients for a health intervention approach and to identify the research gaps for further studies.

## Methods

### Search strategy

This systematic literature review followed the guidelines outlined in the Preferred Reporting Items for Systematic Reviews and Meta-Analyses (PRISMA) 2020 Statement. The search for relevant articles in English, published between 2014 and 2024, was conducted across three indexed databases: Scopus, PubMed, and Web of Science, on February 14th, 2024. The keywords used for the article search are listed in Table 1.

**Table 1:** Keywords used in the screening process.

Database	Search string
Scopus	TITLE-ABS-KEY (("illness perception" OR "perceived illness" OR "perceived health status") AND ("non-communicable disease*" OR "NCD" OR "chronic respiratory disease*" OR "chronic obstructive pulmonary disease*" OR "neoplasm*" OR "cancer" OR "cardiovascular" OR "stroke" OR "heart attack" OR "myocardial infarction" OR "hypertension" OR "diabetes" OR "diabetes mellitus" OR "respiratory illness") AND ("physical activity" OR "exercise" OR "sports" OR "workout"))
PubMed	(("illness perception"[Title/Abstract] OR "perceived illness[title/Abstract] OR "perceived health status[Title/Abstract] AND ("non-communicable disease*") [Title/Abstract] OR "NCD"[Title/Abstract] OR "chronic respiratory disease*[Title/Abstract] OR "chronic obstructive pulmonary disease*"[Title/Abstract] OR "neoplasm*"[Title/Abstract] OR "cancer"[Title/Abstract] OR "cardiovascular"[Title/Abstract] OR "stroke"[Title/Abstract] OR "heart attack[title/Abstract] OR "myocardial infarction"[Title/Abstract] OR "hypertension"[Title/Abstract] OR "diabetes"[Title/Abstract] OR "diabetes mellitus"[Title/Abstract] OR "respiratory illness[Title/Abstract] AND "physical activity[Title/Abstract] OR "exercise"[Title/Abstract] OR "sports"[Title/Abstract] OR "workout"[Title/Abstract])
Web of Science	((TS = ("illness perception" OR "disease perception" OR "health perception")) AND TS = ("non-communicable disease*" OR "NCD" OR "chronic respiratory disease*" OR "chronic obstructive pulmonary disease*" OR "neoplasm*" OR "cancer" OR "cardiovascular" OR "stroke" OR "heart attack" OR "myocardial infarction" OR "hypertension" OR "diabetes" OR "diabetes mellitus" OR "respiratory illness")) AND TS = ("physical activity" OR "exercise" OR "sports" OR "workout")

### Inclusion and exclusion criteria

The inclusion criteria were as follows: (i) Physical activity as the outcome; (ii) Publication in the English language; (iii) Original articles, and (iv) Publication from 2014 to 2024. We limited the publication date to this range (articles published in the past 10 years) to ensure that our systematic review was based on recent literature. Exclusion criteria were: (i) No factor of illness perception exposure; (ii) The term “illness perception” was not properly defined; (iii) Insufficient description of the study method, and (iv) Articles focusing on populations other than NCD patients.

### Eligibility

The initial search identified 585 citations, of which 274 records were excluded based on year, language, and article type using an automated tool. After removing 30 duplicate records, 281 unique records were subjected to title screening by two authors (SMSS and NA). During screening, 250 articles were excluded. The remaining 31 articles underwent eligibility assessment, with disagreements resolved through discussion with a third author (RI) to reach a consensus. Fourteen articles were subsequently removed, two of which focused on non-NCD populations, and twelve did not examine the role of illness perception in the physical activity domain. This left a total of 17 articles for quality appraisal.

### Quality assessment tool

Two authors (SMSS and NA) critically appraised the quality of the included articles independently using the Joanna Briggs Institutes (JBI) Critical Appraisal Tools. The quality assessment was based on the study design checklist [31, 32], and the results are summarised in Table 2.

**Table 2:** Joanna Briggs Institutes Critical Appraisal Tools.

Cross-sectional study design																										
Article No	Article					1		2		3		4		5		6		7		8		Overall (include/exclude)			Comment	
1.	Gu et al 2023 [26]					Y		Y		Y		Y		Y		Y		Y		Y		include				
2.	Li et al 2022 [27]					Y		Y		Y		Y		Y		Y		Y		Y		include				
3.	Lu et al. 2022 [33]					Y		Y		Y		Y		Y		Y		Y		Y		include				
4.	Kwak et al.2022 [34]					Y		Y		Y		Y		Y		Y		N		Y		include			diabetes (HbA1c ≥ 6.5%) and a group with prediabetes (HbA1c 5.7–6.4%)	
5.	Cole et al 2021 [35]					Y		Y		Y		Y		Y		Y		Y		Y		include				
6.	Alyami et al 2020 [29]					Y		Y		Y		Y		Y		Y		Y		Y		include				
7.	Peersen et al [36]					Y		Y		Y		Y		Y		Y		Y		Y		include				
8.	Lan et al. 2019 [37]					Y		Y		Y		Y		Y		N		Y		Y		include				
9.	Forechi et al. 2018 [38]					Y		Y		Y		Y		Y		Y		Y		Y		include				
10.	Nur 2018 [39]					Y		Y		Y		Y		N		N		Y		Y		include			Analysis adequate	
11.	Kugbey et al. 2017 [40]					Y		Y		Y		Y		Y		N		Y		Y		include			Confounding mentioned in the limitation	
12.	Mosleh & Almalik 2014 [41]					Y		Y		Y		Y		Y		Y		Y		Y		include				
13.	Stallings 2015 [42]					Y		Y		Y		Y		Y		Y		Y		Y		include				
14.	Flora et al. 2015 [43]					Y		Y		Y		Y		N		N		Y		Y		include				
15.	Zoeckler et al 2014 [44]					Y		Y		Y		Y		Y		Y		Y		Y		include				
16.	Blair et al 2014 [45]					Y		Y		Y		Y		Y		Y		Y		Y		include				
Randomised Control Trial (RCT) study design																										
Article No	Article	1	2	3	4		5		6		7		8		9		10		11		12		13	Overall (include/exclude)		Comment
17	Rouleau et al 2018 [46]	Y	Y	Y	Y		Y		Y		N		Y		Y		Y		Y		Y		Y	include		RCT

Y = yes, N = No, U = Unclear, NA = Not applicable

### Data extraction tool

**Fig 1:**
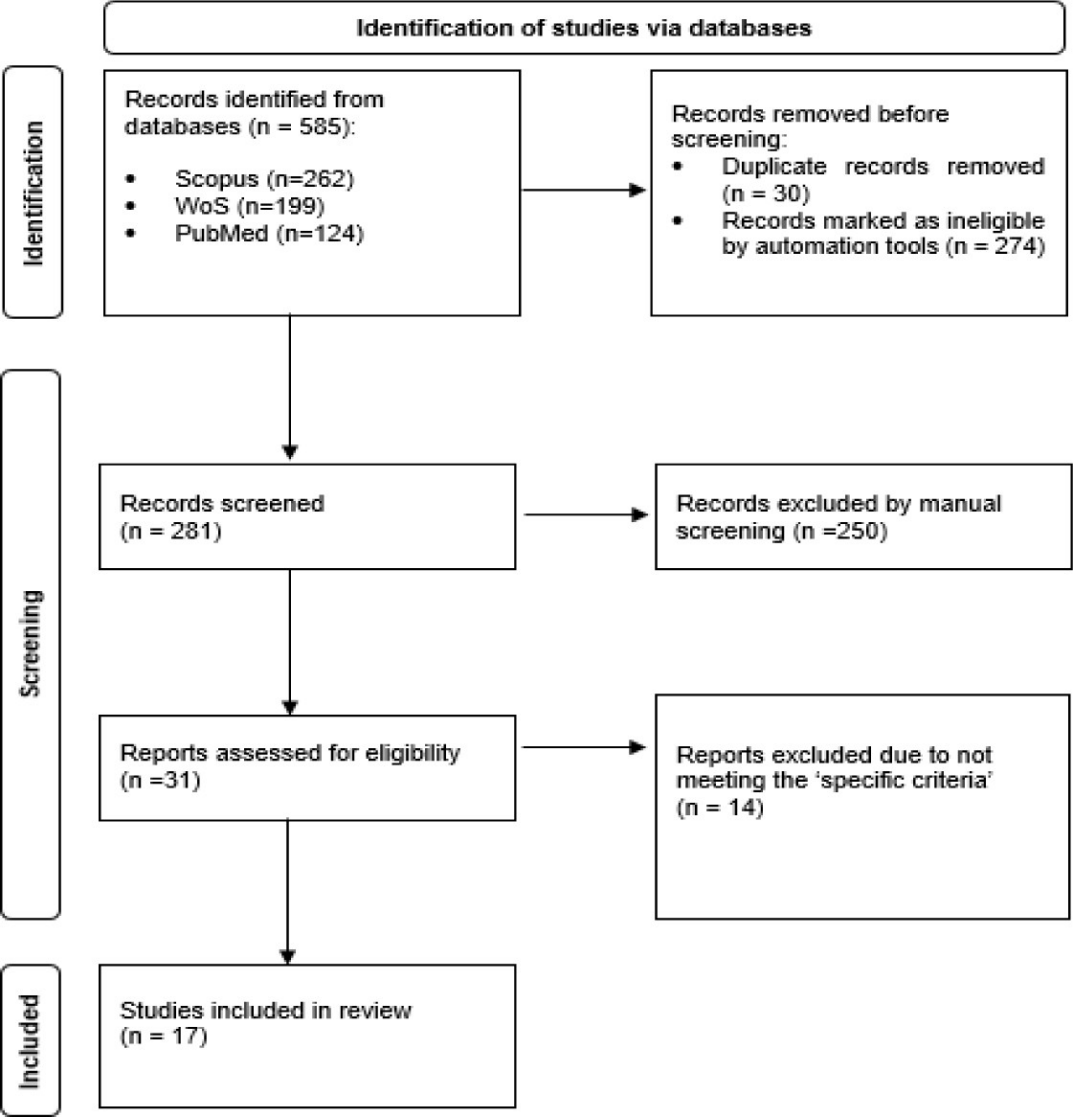
Study selection process according to PRISMA flowchart.

All authors independently extracted information from each article using a standardised Excel spreadsheet, which was then reviewed by the first and second authors. The third author provided additional perspectives and helped resolve any discrepancies between the first and second authors. The extracted information included: (i) author, (ii) publication year, (iii) study design, (iv) country, (v) population, (vi) illness perception measurement tool, (vii) illness perception role, and (viii) statistical analysis. Fig 1 presents the PRISMA flow diagram.

## Results

### Background of the eligible studies

Seventeen studies were included in this systematic review (Table 3). Most of the studies were conducted in China (n = 4), followed by two studies in Canada, with the remaining studies conducted in Australia, Africa, Brazil, Germany, Indonesia, Jordan, Korea, Norway, Saudi Arabia, Scotland, and the United States of America (USA). A total of 10 articles were published between 2014 to 2019, while seven articles were published between 2020 to 2024. The majority of the included studies were cross-sectional studies (n = 16), with one study being a randomised control trial (RCT). The detailed description of the included studies is supplemented in the S3 Table.

**Table 3:** Characteristics of included studies on the role of illness perception.

No	Author, Year	Study Country	NCD	Illness Perception Measurement	Role of Illness Perception a) Direct / Indirect effect by total /dimension/ item b) Significant dimension/item	Physical Activity Measurement	Statistical Analysis
**Cross-Sectional Study Design**							
**Coronary heart disease**							
1.	Peersen et al. 2020 [36]	Norway	n = 1101 Myocardial infarction (MI)	BIPQ	a) Direct effect by dimensions of illness perception b) Significant dimensions: i. Illness control ii. Illness understanding	Self-reported physical activity status by average frequency: i. inactive: < 1 time/week ii. low activity: ≥ 1 time/week and < moderate intensity of 30 minutes ≥ 2–3 times/week iii. adequate activity: ≥ moderate intensity of 30 minutes ≥ 2–3 times/week)	Descriptive, Chi-square test, Analysis of Covariance (ANCOVA), Pearson’s correlation, Multiple linear regression
2.	Nur 2018 [39]	Indonesia	n = 235 Ischaemic heart disease	BIPQ: nine dimensions	a) Direct effect by total illness perception	Modified Cardiac Health Behaviour Scale (CHBS): physical activity dimension	Descriptive, Pearson’s correlation
3.	Mosleh & Almali 2016 [41]	Jordan	n = 254 Coronary heart disease	BIPQ: nine dimensions	a) Direct effect by dimensions of illness perception b) Significant dimensions: i. Personal control ii. Timeline iii. Illness coherence iv. Concern v. Consequences	Godin Leisure-Time Activity Questionnaire (7-day recall on exercise frequency of mild, moderate and strenuous levels that lasted for > 15 minutes)	Descriptive, Chi-square test, Independent t-test, Kruskall–Wallis test, Multiple linear regression
4.	Flora et al. 2015 [43]	Canada	n = 49 Coronary heart disease	IPQ-R: eight dimensions	a) Direct effect by total illness perception	Cardiac rehabilitation participation (total number of minutes per week exercised at the cardiac rehabilitation program)	Descriptive, Analysis of Variance (ANOVA)
5.	Blair et al. 2014 [45]	Scotland	n = 128 Coronary heart disease	BIPQ: nine dimensions	a) Direct effect by total illness perception	Attendance on cardiac rehabilitation session (‘Attenders’ or ‘Non-attenders’)	Descriptive, Dependent t-test, Chi-square test, Multiple logistic regression
**Hypertension**							
6.	Lu et al. 2022 [33]	China	n = 508 Hypertension	Self-perceived disease control question: “Compared to when we interviewed you in R’s LAST IW MONTH, YEAR, is your condition better, about the same as it was then or worse?”	a) Direct effect by total illness perception	Binary answers to the participation of physical activities questions: three questions	Descriptive, Independent t-test, ANOVA, Pearson correlation, Multiple linear regression
7.	Forechi et al. 2018 [38]	Brazil	n = 14,521 • Hypertension • Diabetes mellitus • Dyslipidaemia	Self-perceived health i. Very good/good ii. Fair iii. Very poor/poor	a) Direct effect by dimension of illness perception b) Significant dimensions: i. Fair/poor perceived health status	International Physical Activity Questionnaire (IPAQ): Leisure Time Physical Activity (LTPA) domain	Descriptive, Chi-square tests, Multiple logistic regression
8.	Stallings 2016 [42]	USA	n = 204 Hypertension	IPQ-R: nine dimensions	a) Direct effect by dimension of illness perception b) Significant dimensions: i. Timeline ii. Environmental Cause iii. Emotional representations	7-Day Physical Activity Recall	Descriptive, Pearson’s correlation, Multiple linear regression
**Cancer**							
9.	Gu et al. 2023 [26]	China	n = 218 Lung cancer	Chinese version of BIPQ: eight-dimensions	a) Indirect effect by total illness perception	Exercise Compliance Questionnaire: 15 items	Descriptive, t-test, Chi-square, Pearson’s correlation, Multiple linear regression, Mediation analysis
10.	Li et al. 2022 [27]	China	n = 281 Breast cancer	IPQ-R: nine dimensions	a) Direct positive effect by dimensions of illness perception b) Significant dimensions: i. Timeline acute/chronic ii. Consequence iii. Emotional representation iv. Identity v. Causal-Cancer and treatment factors vi. Causal-Behavioural factors	Functional Exercise Adherence Scale (FEAS): three dimensions (18 items)	Descriptive, Independent t-tests, ANOVA, Pearson’s correlation
11.	Cole et al. 2021 [35]	Australia	n = 366 Cancer	IPQ-R: four dimensions	a) Direct effect by dimensions of illness perception b) Significant dimensions: i. Personal Control ii. Emotional Representation	Godin Leisure-Time Activity questionnaire	Descriptive, Pearson’s correlations, ANCOVA
12.	Lan et al. 2019 [37]	China	n = 124 Breast cancer	The Chinese version of IPQ-R	a) Direct effect by dimensions of illness perception b) Significant dimensions: i. Personal control ii. Treatment control	FEAS	Descriptive, ANOVA, Pearson’s correlation, Multiple linear regression
**Diabetes mellitus**							
13.	Kwak et al. 2022 [34]	Korea	n = 2,485 Prediabetes & Diabetes mellitus	Subjective health status based on a 5-point Likert scale	a) Direct effect by total illness perception	Seven health-related behaviours (Alameda 7): exercise item	Descriptive, Crossover analysis, General linear model
14.	Alyami et al. 2020 [29]	Saudi Arabia	n = 115 Type 2 Diabetes mellitus	BIPQ: nine dimensions	a) Direct effect by dimensions of illness perception b) Significant dimensions: i. Consequences ii. Personal control iii. Treatment control iv. Concerns v. Coherence	Summary of Diabetes Self-Care Activities (SDSCA): exercise dimension (2 items)	Descriptive, Chi-square test, ANOVA, Spearman’s correlation, Multiple linear regression
15.	Kugbey et al. 2017 [40]	Africa	n = 160 Type 2 Diabetes mellitus	BIPQ: nine dimensions	a) Direct effect by total illness perception	Self-care practices questionnaire: exercise domain (two items)	Descriptive, Pearson’s correlation, Multiple linear regression
**COPD**							
16.	Zoeckler et al. 2014 [44]	Germany	n = 96 COPD	IPQ-R: nine dimensions	a) Direct effect by dimensions of illness perception b) Significant dimensions: i. Chronic timeline ii. Emotional representations	6 Minutes Walk Test (6MWT)	Descriptive, Dependent t-test, Chi-square test, Pearson’s correlation, Multiple linear regression
**RCT Study Design**							
17.	Rouleau et al. 2018 [46]	Canada	n = 96 Coronary heart disease	BIPQ: eight dimensions	a) No significant effect	Intention to Attend Cardiac Rehabilitation: two items (seven-point Likert Scale)	Descriptive, ANCOVA, Pearson’s correlation, Multiple logistic regression, Mediation analysis

A total of five types of questionnaires have been used to measure the illness perception variable: the BIPQ, IPQ-R, self-perceived health, self-perceived disease control questionnaire (consisting of three items), and subjective health status. Among these, the BIPQ was the most frequently used, followed by the IPQ-R. Higher scores on both the BIPQ and IPQ-R indicate more negative perceptions towards the disease.

### The roles of illness perception towards physical activity domain among NCD patients

The role of illness perception as a direct and indirect mediator, either by total or its dimension has been demonstrated in the included studies. Majority of the selected articles reported a significant direct effect of illness perception towards the physical activity domain. Only one article demonstrated a significant indirect effect of illness perception between frailty and exercise adherence [26]. The physical activity domain among NCD patients is measured as physical activity determined by a questionnaire [26, 27, 29, 33–35, 37–42], self-reported exercise duration [36, 43], physical activity test [44], cardiac rehabilitation attendance [45], and adherence [46].

#### Coronary heart disease

A total of six selected studies were conducted among patients with coronary heart disease. Five studies demonstrated illness perception’s role as a direct effect [36, 39, 41, 43, 45]. Three of the studies revealed a direct effect of total illness perception [39, 43, 45], and another two studies examined by its dimension [36, 41]. The significant dimensions are personal control, timeline, illness coherence, concern, consequences dimensions [41], illness control, and understanding [36]. Among these five studies, only one reported a positive correlation between total illness perception and physical activity in cardiac health behaviour [39]. However, the sixth study reported a non-significant association between illness perception and physical activity [46].

The list of causal factors obtained from item 9 of the BIPQ was classified as ‘modifiable’ (e.g., diet, weight, lifestyle, smoking, physical inactivity, excessive alcohol, and stress) or ‘non-modifiable’ (e.g., genetic factors, age, gender, and ethnicity) [45]. Patients who attributed their illness to non-modifiable factors were significantly less likely to attend cardiac rehabilitation programmes [45].

#### Hypertension

Three included studies conducted among patients with hypertension demonstrated illness perception’s role as a direct effect [33, 38, 42]. One of the studies demonstrated the significant role of total illness perception and the positive correlation with physical activity [33]. While another two studies reported the significant role of illness perception’s dimensions of fair or poor perceived health status [38], timeline, environmental cause, and emotional representations [42].

#### Cancer

There were four studies carried out among patients with cancer [26, 27, 35, 37]. One of the studies demonstrated total illness perception’s role as an indirect effect [26]. The perception of illness played a partial role as a mediator between frailty and post-operative exercise adherence among patients with lung cancer [26]. While other three studies reported illness perception’s role by its dimensions, namely timeline, consequences, identity, causal-cancer and treatment factors, causal-behaviour factor [27], emotional representations [27, 35], and personal control [35]. A positive correlation was demonstrated between the personal control dimension of illness perception and physical activity adherence among patients with breast cancer [37]. However, a negative correlation was reported between the dimension of treatment control [37] and total illness perception [26] and with physical activity adherence. This means that the higher the illness perception, the worse the exercise adherence [26].

#### Diabetes mellitus

There were three included studies conducted among patients with diabetes mellitus which demonstrated illness perception’s role as direct effect [29, 34, 40]. Two of the studies were conducted among patients with Type 2 Diabetes Mellitus (T2DM) [29, 40]. Another study was conducted among patients with prediabetes and diabetes mellitus without differentiating the types of diabetes mellitus [34]. One of the studies reported the significant role by illness perception dimension of consequences, personal control, treatment control, concerns, and coherence [29]. A study among patients with T2DM at General Hospital in Africa demonstrated a negative correlation between total illness perception with physical activity frequency [40].

#### Chronic obstructive pulmonary disease (COPD)

A study conducted among patients with COPD (Global Initiative for Chronic Obstructive Lung Disease [GOLD] Stage III or IV) demonstrated illness perception’s role as a direct effect of its dimension [44]. The significant illness perception dimensions are chronic timeline and emotional representations [44]. The chronic timeline dimension demonstrated a positive correlation, but the emotional representations dimension showed a negative correlation with physical activity capacity after rehabilitation [44].

## Discussion

Despite the limited literature on the role of illness perception in the physical activity domain among NCD patients, it is evident that illness perception is a significant key factor in this relationship [30]. Among patients with coronary heart disease, the illness perception dimension of illness coherence and understanding played a crucial role in physical activity adherence [36, 41]. The coherence dimension measures heart disease understanding, which can be improved through heart disease literacy, self-efficacy, treatment options, and prognosis [47]. Support and communication with the healthcare provider should also be integrated into the management plan of patients with coronary heart disease to enhance coherence in how the patient perceives their illness. This will help in improving physical activity adherence, specifically through a cardiac rehabilitation programme [48].

The cardiac rehabilitation programme design is critical to improving patient outcomes with individualised and supervised exercise guidance. Despite the established benefits, previous studies carried out among referred cardiac rehabilitation patients revealed that total illness perception scores were higher among individuals who did not attend cardiac rehabilitation programme [43, 45]. This contributes to the fact that patients do not perceive the cardiac rehabilitation programme as potentially beneficial for them [37]. Another critical finding is that the causal belief attribution to the heart disease perceived by the patients was a non-modifiable factor. Therefore, it is less likely that the patient will adopt any form of lifestyle change, specifically physical activity, through a cardiac rehabilitation programme [28]. An effective strategy to align the causal belief attribution is through a patient-centred approach during the consultation period on clinic visit [49].

Among patients with hypertension, a poor or fair illness perception level suggests that a more threatening illness belief could have been addressed during specific interventions or routine hypertension appointment visits to healthcare [38]. Addressing threatening illness beliefs could enhance awareness of the risk associated with hypertension, which can be a motivator for behaviour change and improve physical activity adherence [49]. Previous studies revealed that understanding the severity and consequences of hypertension, specifically in the context of environmental causes, can motivate individuals to engage in physical activity [42, 50]. Examples of environmental causes are environmental pollution, chance or bad luck [42].

The illness perception dimensions of timeline [27], emotional representations [27, 35], treatment control [37], and personal control [35, 37] played a crucial role in physical activity adherence, specifically among patients with cancer. The timeline dimension has an important association with the patient’s treatment control. Patients who perceived cancer as a chronic illness were more likely to have treatment control and adherence compared to those with acute illness perception [28]. Higher treatment control has been associated with greater acceptability of treatment and behaviour intervention [51]. For patients with cancer and COPD, studies have shown that individuals with strong sense of control and positive emotional representations over their illness demonstrated higher physical activity adherence [26, 44, 52]. These perceptions can enhance their belief in the effectiveness and increase their engagement in physical activity adherence [52]. Therefore, it is important to address the patient’s perceptions when designing any physical activity programmes for these patients [26].

The illness perception factor has been reported as a partial mediator of various NCD outcomes, namely diabetes-related distress [53], glycaemic control [54], depression among patients with heart failure [55], and illness-coping among patients with diabetes mellitus [56]. Of the 17 included studies, only one article demonstrated the partial mediator role of illness perception between the frailty of post-thoracoscopic surgery in patients with lung cancer and physical activity adherence [26]. The post-operative condition of chest tube retention and fever worsens the frailty level and affects the patients’ illness perception of illness. Based on the Common-sense Model of illness self-regulation, it is explained that negative coherence and emotion regarding the illness can lead to negative behaviour adoption, like low adherence to physical activity [57].

Among patients with diabetes mellitus, the coherence dimension refers to the perceived understanding and sense of comprehensibility of an individual regarding diabetes mellitus [58]. It will reflect an individual’s coping mechanisms and management strategies. Patients with diabetes mellitus who have a strong sense of coherence are more likely to engage in regular physical activity. This is explained by increasing motivation, reducing a sense of burden, and promoting better self-care practice [58].

This review supported that illness perception should be integrated into NCD patient management. This approach can be implemented through a dedicated health education session targeting newly diagnosed NCD patients conducted by a health educator. The content should be angled according to the specific NCD type which covers the illness perception dimensions. The illness perception role in physical activity adherence can be beneficial through a patient-centred physical activity program designed during sessions conducted with the physiotherapist. These approaches can be implemented via physical or virtual clinics as an individual or group-based program to overcome the healthcare access equity issues faced among NCD patients [59].

This review identified that perceived causal attribution of non-modifiable factors contributed to low physical activity participation and adherence. Therefore, prospective studies on screening of perceived causal attributions may help to identify those who would benefit from early and targeted intervention to increase participation and adherence to physical activity. Future research is also recommended in looking into the effectiveness of the illness perception approach in physical activity adherence programs among NCD patients.

### Strengths and limitations

The majority of the included studies had a cross-sectional design, which directed this systematic review to a high risk of bias and the inability to report causal relationships. Restriction on English-language articles limits the range of article inclusion, which may exclude good-quality articles published in other languages. The use of tools for translation can lead to an increased risk of instrument bias. Nevertheless, the search strategy has resulted in literature sources from various countries where English is not the first language (China, Saudi Arabia, Indonesia).

The strength of this study is the eligibility criteria that only include a clear definition of illness perception towards specific physical activity domains. Additionally, to the best of our knowledge, this is the first systematic review of synthetizing research evidence on the role of illness perception towards the physical activity domain among NCD patients. This systematic review highlights the significance of the illness perception approach in improving physical activity adherence which has been widely reported as the lowest health-promoting lifestyle domain level among various population backgrounds.

### Conclusion

This systematic review triggers the integration of the illness perception dimension into the consultation approach during appointment sessions with NCD patients. The importance of a patient-centred approach in developing a physical activity intervention programme specifically is crucial, as the patients’ illness perception has been proven to play a significant role either as a direct or indirect effect. Significant positive illness perception dimensions like disease timeline, personal control, and patients’ emotions should be promoted. These findings will guide the healthcare provider in developing the management and intervention program according to local settings and population background. Future research should be focused on the effect of interventions tailored to the illness perception approach in order to ensure physical activity participation and adherence continuously improve.

## Supporting information

S1 Table The PRISMA 2020 checklist. (DOCX)

S2 Table The PRISMA 2020 Abstract checklist. (DOCX)

S3 Table Studies identified in literature search. (DOCX)

S4 Table The detailed description of studies included in systematic literature review. (DOCX)
