# Supplementary material for: The role of illness perception in the physical activity domain of health-promoting lifestyle among patients with non-communicable diseases: A systematic review
Source: PLoS One. 2024 Nov 8;19(11):e0311427. doi: 10.1371/journal.pone.0311427 (PMC11548775; doi:10.1371/journal.pone.0311427)
Supplement: S3 Table — (DOCX) [file pone.0311427.s003.docx]

**S3. Studies identified in literature search.**

| No | Title | 1st screener | 2nd screener | 3rd screener | Final status | Reason for exclusion | Authors | Publication Year |
| --- | --- | --- | --- | --- | --- | --- | --- | --- |
|  | Adherence to physical activity in adults with chronic diseases: ELSA-Brasil | include | include |  | include |  | Forechi L, Mill JG, Griep RH, Santos I, Pitanga F, Molina MDCB. | 2018 |
|  | Illness perception, diabetes knowledge and self-care practices among type-2 diabetes patients: a cross-sectional study | include | include |  | include |  | Kugbey N, Oppong Asante K, Adulai K. | 2017 |
|  | The relationship among illness perception, coping and functional exercise adherence in Chinese breast cancer survivors | include | include |  | include |  | Lan M, Zhang L, Zhang Y, Yan J. | 2019 |
|  | Effects of Subjective Health Perception on Health Behavior and Cardiovascular Disease Risk Factors in Patients with Prediabetes and Diabetes | include | exclude | include | include | Classification pre-diabetes and diabetes based on single HbA1c result | Kwak, Sungjung; Lee, Yoonmi; Baek, Seunghui; Shin, Jieun | 2022 |
|  | Illness perception and adherence to healthy behaviour in Jordanian coronary heart disease patients | include | include |  | include |  | Mosleh, Sultan M.; Almalik, Mona M. A. | 2016 |
|  | Illness Perceptions and Adherence to Exercise Therapy in Cardiac | include | include |  | include |  | Parminder K. Flora, Tara J. Anderson, and Lawrence R. Brawley | 2015 |
|  | Interaction between self-perceived disease control and self-management behaviours among Chinese middle-aged and older hypertensive patients: the role of subjective life expectancy | include | include |  | include |  | Lu, Jiao; Liu, Linhui; Zheng, Jiaming; Zhou, Zhongliang | 2022 |
|  | The evaluation of a brief motivational intervention to promote intention to participate in cardiac rehabilitation: A randomized controlled trial | include | include |  | include |  | Rouleau C.R.; King-Shier K.M.; Tomfohr-Madsen L.M.; Bacon S.L.; Aggarwal S.; Arena R.; Campbell T.S. | 2018 |
|  | Medical and Psychosocial Factors Associated with Low Physical Activity and Increasing Exercise Level after a Coronary Event | include | include |  | include |  | Peersen K.; Otterstad J.E.; Sverre E.; Perk J.; Gullestad L.; Moum T.; Dammen T.; Munkhaugen J. | 2020 |
|  | The role of illness perceptions and exercise beliefs in exercise engagement during treatment for cancer | include | include |  | include |  | Cole S.F.; Skaczkowski G.; Wilson C. | 2021 |
|  | Health literacy for cardiac rehabilitation: An examination of associated illness perceptions, self-efficacy, motivation and physical activity | include | exclude | exclude | exclude | Looking relation between health literacy with illness perception and exercise | Walters R.; Leslie S.J.; Sixsmith J.; Gorely T. | 2020 |
|  | Frailty, Illness Perception and Lung Functional Exercise Adherence in Lung Cancer Patients After Thoracoscopic Surgery | include | include |  | include |  | Gu X.; Shen X.; Chu J.-H.; Fang T.-T.; Jiang L. | 2023 |
|  | Illness perceptions and adherence to breast cancer-related lymphedema risk management behaviours among breast cancer survivors | include | include |  | include |  | Li M.; Huang W.; Zhang X.; Chen J.; Luo X.; Zhang Y.; Xiong C.; Yan J. | 2022 |
|  | The association of illness perceptions and God locus of health control with self-care behaviours in patients with type 2 diabetes in Saudi Arabia | include | include |  | include |  | Alyami M.; Serlachius A.; Mokhtar I.; Broadbent E. | 2020 |
|  | Illness perceptions predict exercise capacity and psychological well-being after pulmonary rehabilitation in COPD patients | include | include |  | include |  | Zoeckler N.; Kenn K.; Kuehl K.; Stenzel N.; Rief W. | 2014 |
|  | Illness perceptions and health behaviors of black women | include | include |  | include |  | Stallings D.T. | 2016 |
|  | The influence of non-modifiable illness perceptions on attendance at cardiac rehabilitation | include | include |  | include |  | Blair J.; Angus N.J.; Lauder W.J.; Atherton I.; Evans J.; Leslie S.J. | 2014 |
|  | Illness perception and cardiovascular health behaviour among persons with ischemic heart disease in Indonesia | include | include |  | include |  | Nur, Kholid Rosyidi Muhammad (57201796540) | 2018 |
|  | The association between subjective health perception and lifestyle factors in Shiga prefecture, Japan: a cross-sectional study | exclude | exclude |  | exclude | Population: general population | Tanaka, Sae; Muraki, Sayu; Inoue, Yuri; Miura, Katsuyuki; Imai, Eri | 2020 |
|  | Biased health perceptions and risky health behaviors-Theory and evidence | exclude | exclude |  | exclude | Exposure is biased health perception | Arni, Patrick; Dragone, Davide; Goette, Lorenz; Ziebarth, Nicolas R. | 2021 |
|  | Predictive Model of Functional Exercise Compliance of Patients with Breast Cancer Based on Decision Tree | exclude | exclude |  | exclude | Exposure is perceived benefit not illness perception | Luo, Zebing; Luo, Baolin; Wang, Peiru; Wu, Jinhua; Chen, Chujun; Guo, Zhijun; Wang, Yiru | 2023 |
|  | Illness perceptions and diabetes self-care behaviours in Maori and New Zealand Europeans with type 2 diabetes mellitus: a cross-sectional study | exclude | exclude |  | exclude | Study explore differences of illness perception and self-care behaviour between Maori and NZ population. Not study the role of IP | Romana J.; Law M.; Murphy R.; Morunga E.; Broadbent E. | 2022 |
|  | Determinants of information needs in patients with coronary artery disease receiving cardiac rehabilitation: a prospective observational study | exclude | exclude |  | exclude | Study explores on patients' need on information but not the illness perception assessment | Tenbult N.; Asten I.V.; Traa S.; Brouwers R.W.M.; Spee R.F.; Lu Y.; Brini A.; Kop W.; Kemps H. | 2023 |
|  | Inpatient Step Counts, Symptom Severity, and Perceived Health Status after Lung Resection Surgery | exclude | exclude |  | exclude | Exposure is perceived health status but not the illness perception | Rodr¡guez-Torres J.; L¢pez-L¢pez L.; Cabrera-Martos I.; Quero-Valenzuela F.; Cahalin L.P.; Cebri…-I-Iranzo M.D.?.; Valenza M.C. | 2021 |
|  | The Relationship between Self-Perceived Health and Physical Activity in the Mental Health of Korean Cancer Survivors | exclude | exclude |  | exclude | IV= self-perceived health & exercise DV=mental health | Kwak S.; Shin J.; Kim J.-Y. | 2023 |
|  | Impact of Early Exercise-based Cardiac Rehabilitation on Hostility, its Behavioral Components and Disease Perception in Patients after Myocardial Infarction | exclude | exclude |  | exclude | IV= exercise DV= disease perception | Korzeniowska-Kubacka I.; Mierzy?ska A.; Rydzewska E.; Smolis E.; D?browski R. | 2023 |
|  | Nonlinear, Multicomponent Physical Exercise with Heart Rate Variability-Guided Prescription in Women with Breast Cancer During Treatment: Feasibility and Preliminary Results (ATOPE Study) | exclude | exclude |  | exclude | No exposure of IP | Gonz lez-Santos ?.; Lopez-Garzon M.; Gil-Guti‚rrez R.; del Mar Salinas-Asensio M.; Postigo-Martin P.; Cantarero-Villanueva I. | 2023 |
|  | Effectiveness of a self-management support program for type 2 diabetes patients in the first years of illness: Results from a randomized controlled trial | exclude | exclude |  | exclude | Effectiveness of interventions towards illness perception and exercise diomain of self-care | Van Puffelen A.L.; Rijken M.; Heijmans M.J.W.M.; Nijpels G.; Schellevis F.G. | 2018 |
|  | The role of illness schemata in self-care behaviors and glycemic control among patients with type 2 diabetes in Iran | exclude | exclude |  | exclude | Relation between IP and exercise with illness schemata | Shibayama T.; Tanha S.; Abe Y.; Haginoya H.; Rajab A.; Hidaka K. | 2019 |
|  | Minimal-Resource Home Exercise Program Improves Activities of Daily Living, Perceived Health Status, and Shortness of Breath in Individuals with COPD Stages GOLD II to IV | exclude | exclude |  | exclude | IV=home exercise DV=health perception | Ribeiro Mo‡o V.J.; Gulart A.A.; Lopes A.J.; de S  Ferreira A.; da Fonseca Reis L.F. | 2023 |
|  | Longitudinal study of the relationship between patients' medication adherence and quality of life outcomes and illness perceptions and beliefs about cardiac rehabilitation | exclude | exclude |  | exclude | IV=IP DV=medication adherence | Thomson P.; Rushworth G.F.; Andreis F.; Angus N.J.; Mohan A.R.; Leslie S.J. | 2020 |
