# Supplementary material for: The role of illness perception in the physical activity domain of health-promoting lifestyle among patients with non-communicable diseases: A systematic review
Source: PLoS One. 2024 Nov 8;19(11):e0311427. doi: 10.1371/journal.pone.0311427 (PMC11548775; doi:10.1371/journal.pone.0311427)
Supplement: S4 Table — (DOCX) [file pone.0311427.s004.docx]

**S****4: The detailed description of studies included in systematic literature review.**

| No | Author, Year | Country | Population | n | Purpose | Outcome | Conclusion | Extractors | Date |
| --- | --- | --- | --- | --- | --- | --- | --- | --- | --- |
|  | Peersen et al. 2020 [36] | Norway | Hospitalised patients with myocardial infarction (MI)  Mean (SD) age: 61.6 (9.6) years | 1101 | Aimed to identify medical and psychosocial factors (e.g: illness perception) associated with physical activity status | Illness perceptions were significantly associated with self-reported increasing physical activity levels. | Further research on the effect of interventions tailored to the illness perception is needed to improve physical activity levels in coronary heart disease patients. | i. SMSS  ii.NA  iii.RI | 20^th^ February 2024 till 1^st^ March 2024 |
|  | Nur 2018 [39] | Indonesia | Patients with ischaemic heart disease  Age (Min-Max: 50-85) years | 235 | Aimed to explore the:   1. level of illness perception 2. level of cardiovascular health behaviour (physical activity dimension) 3. relationship between illness perception and cardiovascular health behaviour | 1. Mean (SD) score of illness perception = 43.65 (3.93) [moderate level]. 2. Mean (SD) score of cardiovascular health behaviour = 80.29 (5.4) [high level]. 3. A significant positive relationship existed between illness perception and cardiovascular health behaviour. | Persons with higher illness perception demonstrated a positive correlation with higher cardiovascular health behaviour | i. SMSS  ii.NA  iii.RI | 20^th^ February 2024 till 1^st^ March 2024 |
|  | Mosleh & Almali 2016 [41] | Jordan | Patients with coronary heart disease  Mean (SD) age: 52.0 (15.6) years | 254 | Aimed to explore the illness perception of patients with coronary heart disease and investigate whether these beliefs could predict adherence to healthy behaviours (physical exercise). | Physical exercise adherence was predicted by a strong perception of personal control, timeline and illness coherence. | Illness perception dimensions like consequences, timeline, personal and treatment control, concern and comprehensive, are important in developing an intervention strategy to motivate and enhance long-term physical exercise adherence. | i. SMSS  ii.NA  iii.RI | 20^th^ February 2024 till 1^st^ March 2024 |
|  | Flora et al. 2015 [43] | Canada | Patients with coronary heart disease    Mean age: NA | 49 | Aimed to examine different illness perception groups among cardiac rehabilitation exercise participants following 3 months of exercise therapy | The strong illness perception group reported significantly lower adherence to cardiac rehabilitation exercises following three months. | Individual illness perception differences relative to exercise therapy adherence may assist in understanding exercise adherence among cardiac rehabilitation participants. | i. SMSS  ii.NA  iii.RI | 20^th^ February 2024 till 1^st^ March 2024 |
|  | Blair et al. 2014 [45] | Scotland | Patients with coronary heart disease referred for cardiac rehabilitation  Mean (SD) age of responders: 65.0 (10.0) years  Mean (SD) age of non-responders: 64.0 (11.0) years | 128 | Aimed to examine the influence of illness perceptions on patient attendance at cardiac rehabilitation. | 1. Non-attendees of cardiac rehabilitation reported higher total illness perception scores. 2. Those who attributed their illness to non-modifiable factors were significantly less likely to attend cardiac rehabilitation. | Early screening of perceived causal attributions may help to identify those who would benefit from early and targeted intervention to increase participation in cardiac rehabilitation. | i. SMSS  ii.NA  iii.RI | 20^th^ February 2024 till 1^st^ March 2024 |
|  | Lu et al. 2022 [33] | China | Participants of China Health and Retirement Longitudinal Survey (CHARLS)  Mean age: NA | 508 | Aimed to examine the relationship between self-perceived disease control and self-management behaviours (physical activity) in Chinese middle-aged and older hypertensive patients. | Good self-perceived disease control predicted good physical activity. | Interventions aimed at enhancing the effect perception of general self-management behaviours on the present disease control perspective, and future lifespan perspective would be beneficial for the consistent self-management behaviours among Chinese middle-aged and older patients with hypertension. | i. SMSS  ii.NA  iii.RI | 20^th^ February 2024 till 1^st^ March 2024 |
|  | Forechi et al. 2018 [38] | Brazil | Patients with hypertension, diabetes mellitus & dyslipidaemia  from the study ELSA-Brasil  Mean age: NA | 14,521 | Aimed to investigate the adherence and the factors that influence adherence to physical activity | 1. 17.8%, 15.1%, and 13.9% of the subjects who reported dyslipidemia, hypertension, and diabetes, respectively, adhere to the physical activity recommendations. 2. Older individuals who reported poor perceived health demonstrated lower adherence to physical activity. | 1. The number of adults with dyslipidemia, hypertension, and diabetes who adhere to the physical activity recommendations is very low. 2. Negative health perception was considered a barrier to physical activity. | i. SMSS  ii.NA  iii.RI | 20^th^ February 2024 till 1^st^ March 2024 |
|  | Stallings 2016 [42] | USA | Patients with hypertension  Mean age:NA | 204 | Aimed to explore the relationships between hypertension representations and exercise. | 1. Perceiving hypertension as chronic and perceiving it because of environmental causes was inversely related to moderate-intensity physical activity. 2. Having more emotional responses to hypertension was related to moderate-intensity physical activity. | Hypertension representations can interfere with recommended lifestyle changes. | i. SMSS  ii.NA  iii.RI | 20^th^ February 2024 till 1^st^ March 2024 |
|  | Gu et al. 2023 [26] | China | Patients with  lung cancer  Mean age: NA | 218 | Aimed to explore the correlation between disease perception and lung functional exercise compliance. | 1. Disease perception was significantly associated with pulmonary function exercise compliance (after controlling for confounding factors) 2. The higher the disease perception negative score, the less exercise compliance demonstrated. 3. Illness perception played a partially mediating role in the association between frailty and lung functional exercise compliance. | 1. Disease perception has an impact on exercise adherence 2. There is a need to consider disease perception in the intervention to improve exercise compliance after thoracoscopic surgery for lung cancer. | i. SMSS  ii.NA  iii.RI | 20^th^ February 2024 till 1^st^ March 2024 |
|  | Li et al. 2022 [27] | China | Postoperative  breast cancer patients in the ward at Guangzhou Hospital  Mean (SD) age: 48.46 (9.47) years | 281 | Aimed to describe illness perceptions of breast cancer-related lymphedema (BCRL) and adherence to BCRL risk management behaviours (e.g: perform appropriate functional exercises of the affected arm every day) | Dimensions of illness perceptions, such as consequence, timeline acute/chronic, illness coherence and identity were positively related to BCRL risk management behaviours adherence. | Illness perceptions are important for BCRL risk management behaviour adherence. | i. SMSS  ii.NA  iii.RI | 20^th^ February 2024 till 1^st^ March 2024 |
|  | Cole et al. 2021 [35] | Australia | Patients with  cancer in oncology & radiotherapy day | 366 | Aimed to examine whether exercise beliefs and illness perceptions were associated with changes in exercise behaviour following a cancer diagnosis  (Main outcome measures are symptom severity, pre- and post-morbid exercise levels, exercise beliefs, and illness perceptions.) | - Pre- and post-morbid exercise levels  1. Decreasers participation (58.1%) 2. Increased participation (30.4%) 3. Maintained (9.2%)   After controlling for symptom severity and time since cancer diagnosis, results demonstrated as below:   - Decreasers compared to Increasers reported as:  1. Lower self-efficacy for exercise 2. Higher levels of belief in the Negative Impact on Cancer of exercise 3. Lower levels of Personal Control 4. Less Emotional Representation of their illness | Identifying unhelpful beliefs about the relationship between exercise and illness during cancer treatment and conducting early intervention could be an effective strategy for preventing cancer patients from decreasing exercise following their diagnosis. | i. SMSS  ii.NA  iii.RI | 20^th^ February 2024 till 1^st^ March 2024 |
|  | Lan et al. 2019 [37] | China | Patients with  breast cancer at Guangzhou Cancer Centre  Mean age: NA | 124 | Aimed to explore functional exercise adherence and illness perception. | The personal control dimension of illness perception was related to functional exercise adherence. | Interventions that improve illness perceptions may lead to better functional exercise adherence among patients with breast cancer. | i. SMSS  ii.NA  iii.RI | 20^th^ February 2024 till 1^st^ March 2024 |
|  | Kwak et al. 2022 [34] | Korea | Participants with pre-diabetic and diabetic from 7^th^ and 8^th^ National Health and Nutrition Survey  Mean age: NA | 2,485 | Aimed to confirm the health behaviour performance (exercise) rate according to the subjective health perception. | The better the subjective health status, the higher the ratio of health-related behaviours. | It is necessary to improve the positive subjective perception of health for disease prevention and continuous management through healthy behaviours. | i. SMSS  ii.NA  iii.RI | 1^st^ -5^th^ March 2024 |
|  | Alyami et al. 2020 [29] | Saudi  Arabia | Patients with  Type 2 Diabetes mellitus at outpatient diabetes centre in Najran, Saudi Arabia  Mean (SD) age: 56.0 (12.43) years | 115 | Aimed to investigate the associations between illness perceptions and self-care behaviours (exercise). | A better understanding of Type 2 Diabetes Mellitus was a significant independent predictor for exercise. | Greater perceptions of personal control and coherence were associated with higher adherence to self-care behaviours. | i. SMSS  ii.NA  iii.RI | 20^th^ February 2024 till 1^st^ March 2024 |
|  | Kugbey et al. 2017 [40] | Africa | Patients with  Type 2 Diabetes mellitus at General Hospital in Accra  Mean (SD) age: 60.3 (12.04) years | 160 | Aimed to examine whether Type 2 Diabetes Mellitus patients’ illness perception significantly predicts diabetes self-care practices (exercise). | The exercise was significantly predicted by illness perception. | Cognitive and emotional representation of diabetes are key determinants for diabetes self-care practices. | i. SMSS  ii.NA  iii.RI | 20^th^ February 2024 till 1^st^ March 2024 |
|  | Zoeckler et al. 2014 [44] | Germany | Patients with  Chronic Obstructive pulmonary Disease (COPD) [GOLD III/IV]  Mean (SD) age: 61.2 (8.8) years | 96 | Aimed to investigate whether illness perceptions before pulmonary rehabilitation influence exercise capacity. | Illness perceptions before rehabilitation predicted exercise capacity at the end of treatment. | It is relevant to identify and change maladaptive illness perceptions to improve outcomes in COPD. | i. SMSS  ii.NA  iii.RI | 20^th^ February 2024 till 1^st^ March 2024 |
|  | Rouleau et al. 2018 [46] | Canada | Patients with  coronary heart disease referred for a centralized cardiac rehabilitation program  Mean (range) age: 59 (34-80) years | 96 | Aimed to investigate the impact of motivational interviews on intention (illness perception) to participate in cardiac rehabilitation. | The illness perception demonstrated a non-significant impact of motivational interviews between the intervention and control groups. | The findings will inform the efforts to design behavioural interventions to enhance cardiac rehabilitation participation. | i. SMSS  ii.NA  iii.RI | 20^th^ February 2024 till 1^st^ March 2024 |

*SMSS= Sharifah Maziah Syed Shamsuddin, NA= Norfazilah Ahmad, RI= Roszita Ibrahim
